# Supplementary material for: Assessment of Scalable Fractionation Methodologies to Produce Concentrated Lauric Acid from Black Soldier Fly (Hermetia illucens) Larvae Fat
Source: Insects. 2025 Feb 6;16(2):171. doi: 10.3390/insects16020171 (PMC11857038; doi:10.3390/insects16020171)
Supplement: Supplementary file 1 [file insects-16-00171-s001.zip › insects-3352771-supplementary.pdf]

Supplementary Material

Assessment of Scalable Fractionation Methodologies to Produce Concentrated Lauric Acid from Black Soldier Fly (*Hermetia illucens*) Larvae Fat

Luis Vázquez [1,2,*], Carlota Reyero [1,2], Raúl Hurtado-Ribeira [1,2], David Villanueva-Bermejo [1,2], Alejandro Belinchón [3], José Palomar [3], Tiziana Fornari [1,2] and Diana Martín [1,2]

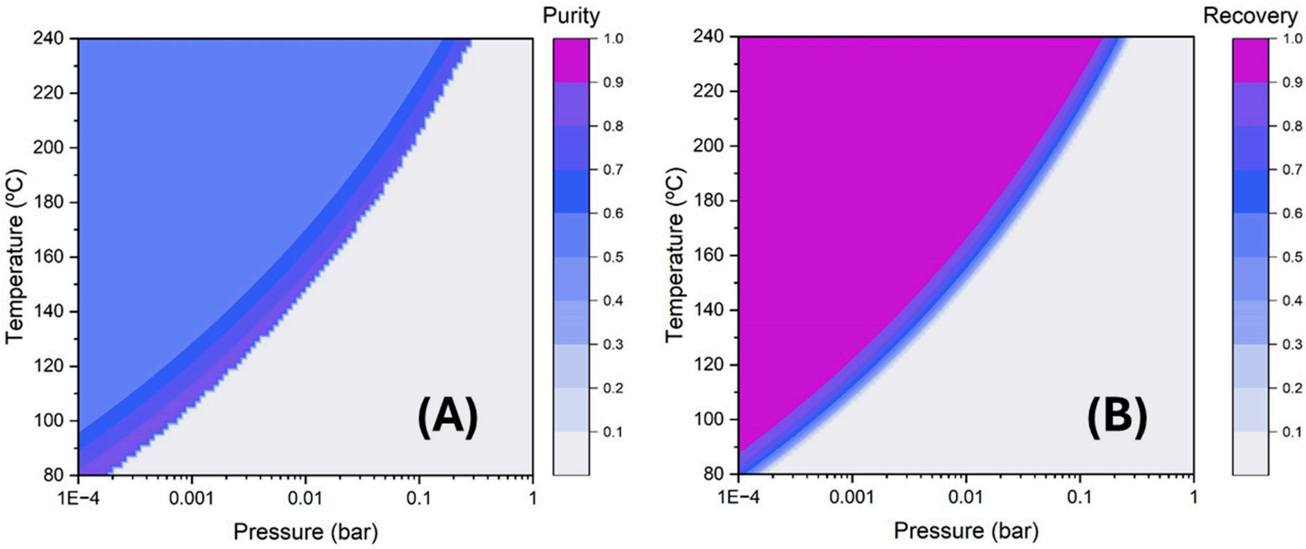

Figure S1. (A) Mass purity and (B) recovery of ethyl laurate acid in the vapor stream employing a simple distillation.

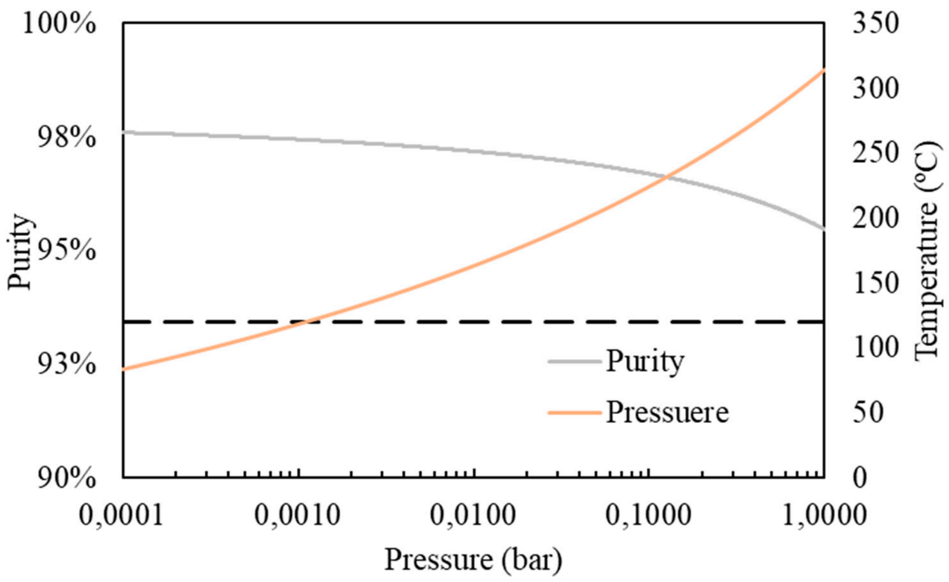

Figure S2. Product mass purity, reboiler temperature and pressure of the 10-stage distillation column are maintained with a constant 80% ethyl laurate recovery.
